# Nanobrick Wall Multilayer Thin Films with High Dielectric Breakdown Strength

**DOI:** 10.1021/acsaenm.3c00439

**Published:** 2023-08-18

**Authors:** Ethan T. Iverson, Hudson Legendre, Shubham V. Chavan, Anil Aryal, Maninderjeet Singh, Sourav Chakravarty, Kendra Schmieg, Hsu-Cheng Chiang, Patrick J. Shamberger, Alamgir Karim, Jaime C. Grunlan

**Affiliations:** † Department of Chemistry, Texas A&M University, College Station, Texas 77843, United States; ‡ Department of Mechanical Engineering, Texas A&M University, College Station, Texas 77843, United States; § Department of Chemical and Biomolecular Engineering, University of Houston, Houston, Texas 77204, United States; ∥ Department of Materials Science and Engineering, Texas A&M University, College Station, Texas 77843, United States

**Keywords:** vermiculite, boehmite clay, thermal conductivity, dielectric breakdown strength, layer-by-layer assembly

## Abstract

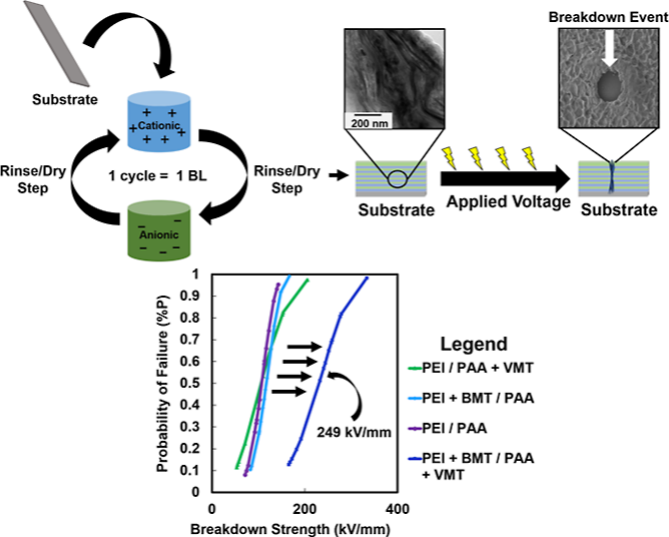

Current thermally conductive and electrically insulating insulation systems are struggling to meet the needs of modern electronics due to increasing heat generation and power densities. Little research has focused on creating insulation systems that excel at both dissipating heat and withstanding high voltages (i.e., have both high thermal conductivity and a high breakdown strength). Herein, a polyelectrolyte-based multilayer nanocomposite is demonstrated to be a thermally conductive high-voltage insulation. Through inclusion of both boehmite and vermiculite clay, the breakdown strength of the nanocomposite was increased by ≈115%. It was also found that this unique nanocomposite has an increase in its breakdown strength, modulus, and hydrophobicity when exposed to elevated temperatures. This readily scalable insulation exhibits a remarkable combination of breakdown strength (250 kV/mm) and thermal conductivity (0.16 W m^–1^ K^–1^) for a polyelectrolyte-based nanocomposite. This dual clay insulation is a step toward meeting the needs of the next generation of high-performance insulation systems.

## Introduction

Various high-voltage electronics for aerospace, defense, and energy storage and conversion have experienced a significant increase in complexity, power draw, and heat generation.^1^ This rapid development has resulted in a significant amount of research focusing on creating and improving materials that can be used as electrical insulation, energy storage devices, thermal management systems, and combinations thereof.^2^ One critical sector of research focuses on a new generation of dielectrics that have higher dielectric breakdown strengths, as well as improved through-plane thermal conductivity.^3^ This is driven by the rapid miniaturization and increasing power draw (i.e., higher operating voltages) of high-voltage electronics, that in combination, results in larger amounts of heat generation. While these efforts have made acceptable strides, breakthroughs in this field are impeded by the inverse relationship between the material’s thermal conductivity and breakdown strength.

Through-plane thermal conductivity of electrical insulation is becoming a prominent property to optimize as modern technology produces far more heat due to higher power densities, power draws, and decreased thermal capacitance.^2^ With these elevated temperatures, insulation can experience thermal breakdown, which occurs when the material is subjected to temperatures above its operation limits, leading to thermal runaway and potential catastrophic electrical failure.^4^ While state-of-the-art technology will reduce power density and power draw when temperatures get too high, thermal runaway still occurs.^5^ Another approach, commonly employed in aviation and electronics for limiting dielectric materials’ temperature exposure, is implementing a cooling system. While this approach historically has been successful, it adds (1) unwanted weight that decreases power density, and (2) can significantly increase the cost.^6^ In tandem with power throttling and cooling systems, more emphasis should be placed on the design of thermal management systems at a molecular level (i.e., chemical composition) so that the through-plane thermal conductivity can be increased. By increasing through-plane thermal conductivity, more heat can be dissipated, which allows for higher operating temperatures (due to lower risk of thermal runaway) and higher power densities and power draws.^1^ If the dielectric material suffers from high dielectric loss that results in electrical energy’s transformation to thermal energy (ultimately adding to the systems’ heat generation), higher thermal conductivity will dissipate the heat and decrease the likelihood of thermal runaway.

While polymer-based dielectrics provide outstanding properties such as high dielectric breakdown strength (>200 kV/mm), low losses, and high dielectric constants, they are plagued with extremely low through-plane thermal conductivity.^2^ Polymer nanocomposites offer an attractive solution by adding thermally conductive inorganics such as aluminum oxides, boron nitride, and silicon nitride, thereby increasing the materials’ thermal conductivity.^7^ However, traditional polymer nanocomposites prepared through blending, co-extrusion, and shear mixing suffer from particle aggregation at high loadings.^8^ Huang et al. presented a nanofiber/boron nitride nanosheet paper-nanocomposite, which led to a staggering combination of a breakdown strength of 440 kV/mm and a thermal conductivity of 22 W m^–1^ K^–1^.^9^ While this paper-nanocomposite displays remarkable properties, its preparation is complex, time-consuming, and difficult to apply to substrates with complex three-dimensional geometries.

Polyelectrolyte-based dielectrics have long been investigated for their use in pressure sensing organic transistors owing to their low-voltage operation and high charge-carrier densities.^10^ In recent years, however, polyelectrolytes have gained attention in the field of insulating thin-film dielectrics due to their ease of processing and low costs. Che et al. prepared a polyelectrolyte-based dielectric material comprising polyvinylidene fluoride latex and chitosan that demonstrated a remarkable breakdown strength and energy density of 630 kV/mm and 10.1 J/cm^3^, respectively.^11^ One drawback of this work is that these properties are only achievable after exposure to high pressures (30 MPa), which could limit thickness options and prove catastrophic for substrates with complex geometries. To combat the issue of conformability and limited thickness options, we previously investigated multilayer dielectrics utilizing layer-by-layer (LbL) assembly of polyelectrolytes and nanoplatelets to demonstrate proof-of-concept.^12,13^

LbL assembly is believed to hold great promise in the realm of dielectrics due to its near perfect conformability on surfaces of any topography, film thickness scalability, and its multilayer composition, which is known to enhance breakdown strength and polarization.^14,15^ LbL processing typically consists of exposing a charged substrate to aqueous solutions of cationic and anionic materials in an alternating fashion. These solutions can consist of charged polymers (i.e., polyelectrolytes), small molecules, nanoplatelets, or a combination thereof. This coating technique can be employed industrially via roll-to-roll processing or spray coating.^16,17^ LbL-assembled nanocomposites most commonly are employed as gas barrier, heat shielding, and/or corrosion protection treatments due to their high inorganic loading (≥70 wt %).^18,19^ Furthermore, LbL nanocomposites are attractive due to their ambient processing conditions and ability to be applied to a variety of substrates. It is believed that these LbL films, containing a tortuous path created by nanoplatelets, will greatly help improve the dielectric properties if loaded with electrically insulating fillers. While LbL assembly of polyelectrolyte-based composites has been investigated for dielectrics and thermal conductivity independently,^12,13,20^ a nanocomposite created to exploit both of these properties has yet to be investigated.

In the present study, the thermal conductivity and dielectric properties of a dual clay nanocomposite are investigated at room temperature and elevated temperatures (simulating operation conditions). Through the inclusion of both boehmite and vermiculite clay, the breakdown strength is increased ∼110% when compared to the polyethyleneimine/poly(acrylic acid) matrix. It was found that the dielectric breakdown strength and thermal conductivity of the nanocomposite is approximately 250 kV/mm and 0.16 W m^–1^ K^–1^, respectively, a combination comparable to that of Kapton. Additionally, it was found that with elevated temperatures, the dielectric breakdown strength of the nanocomposite increases, likely due to expulsion of molecular water. This study evaluates, for the first time, a LbL-generated nanocomposite that possesses a difficult to achieve combination of through-plane thermal conductivity and dielectric breakdown strength. These findings should spur further development of thermally conductive yet electrically insulating nanocomposites that will protect high-voltage electronics.

## Experimental Section

### Materials

Branched polyethylenimine (PEI, *M*_w_ = 25 kg/mol) and poly(acrylic acid) (PAA, *M*_w_ = 250 kg/mol in a 35 wt % aqueous solution) were purchased from Sigma-Aldrich (Milwaukee, WI, USA). Microlite 963++ vermiculite clay (VMT, 7.8 wt % aqueous solution) was purchased from Specialty Vermiculite Corp. (Cambridge, MA, USA) and boehmite clay (BMT) was purchased from Esprix Technologies (Sarasota, FL, USA). All molecular weight information was obtained from chemical suppliers and chemicals and clays were used without further manipulation. Aqueous solutions and rinses utilized 18 MΩ deionized (DI) water. All solutions were composed of a mixture of polymer and clay. Cationic solutions were prepared as 0.1 wt % PEI + 0.5 wt % BMT aqueous solutions. The PEI + BMT solution was rolled for 24 h to ensure homogeneous dispersion, after which the pH was determined to be ≈9. Previous reports have determined BMT to have an average characteristic length of 180 nm, in aqueous dispersions, and a hexagonal schistose shape.^21^ Anionic solutions were prepared as 0.1 wt % PAA + 1 wt % VMT aqueous solutions. The PAA + VMT solution was rolled for 24 h to ensure homogeneous dispersion, after which the pH was determined to be ≈5. Previous reports have determined VMT to be a magnesium-aluminum-silicate with an average effective diameter of 1.1 μm and a density of 1.05 g/cm^3^.^21^ Indium-tin-oxide (ITO)-coated glass slides as well as polished 500 μm-thick undoped silicon wafers with a resistance of 10,000 Ω cm were purchased from University Wafer (South Boston, MA, USA). All substrates were rinsed in DI water, followed by an ethanol rinse, and then another DI water rinse. Substrates were then dried with compressed filtered air and subjected to a 5 min plasma cleaning utilizing an ATTO plasma cleaner (Diener Electronic, Ebhausen, Germany).

### Preparation of Nanocomposites

**Figure 1:**
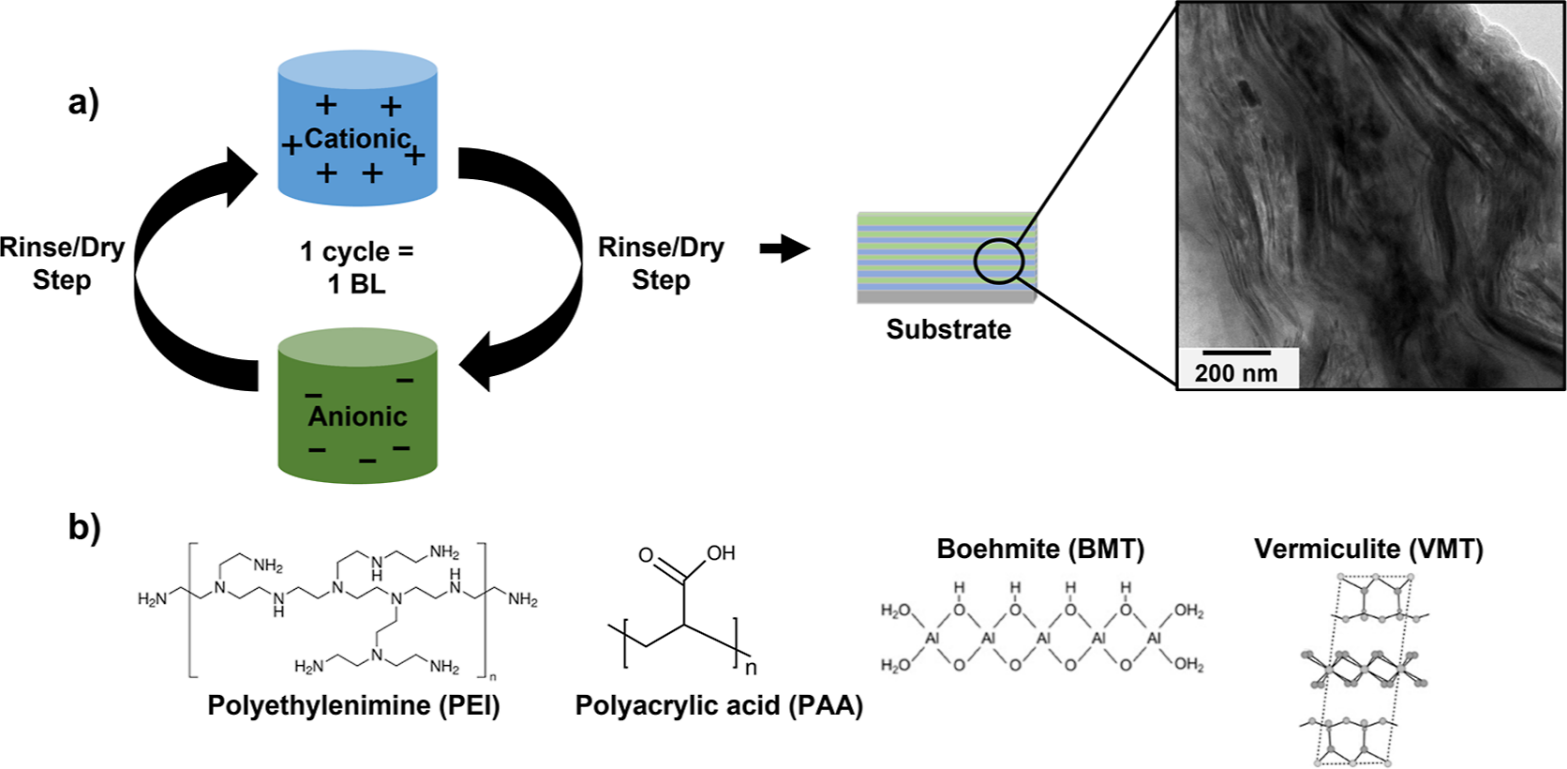
(a) Schematic of the LbL deposition process and cross-sectional TEM image of the nanocomposite. (b) Chemical structures of PEI, PAA, BMT, and VMT.

Nanocomposites were grown by first dipping plasma-treated substrates into the cationic (PEI + BMT) solution for 5 min followed by a DI water wash and blown dry with a filtered air blade to remove any loosely adhered material. The substrate was then submerged into the anionic (PAA + VMT) solution for 5 min, followed by a DI water wash and blown dry with a filtered air blade to remove any loosely adhered material. This cycle completed the first bilayer (BL), after which all subsequent BL were deposited in a similar fashion, except the dip time was reduced to 1 min. The PEI + BMT/PAA and PEI/PAA + VMT control nanocomposites were prepared in the same manner, but one of the solutions contained only polymers (either PEI or PAA) depending on the nanocomposite. For the PEI/PAA control, the composite was also prepared in the same manner, but each solution contained only polymer. The deposition cycle, chemical structures, as well as a cross-sectional transmission electron microscopy (TEM) micrograph are displayed in Figure 1. Initial dip times were longer for the first BL to ensure uniform substrate coverage during the initial BL deposition. The PEI + BMT/PAA + VMT nanocomposite growth curve is shown in Figure S1.

### Nanocomposite Characterization

Prior to all characterization, nanocomposites were stored in a dry box for approximately 24 h. Nanocomposite thickness and surface roughness values (*R*_A_ and *R*_Q_) were measured utilizing a KLA-Tencor P-6 Stylus Profiler (Milpitas, CA, USA) or an Alpha-SE ellipsometer (J.A. Woollam Co., Lincoln, NE, USA) depending on film thickness. Each sample had its average thickness and roughness tabulated in triplicate. The absence of crosslinking immediately before and after elevated temperature exposure was confirmed by scraping the nanocomposites off of the substrate and subjecting the powdered film to Fourier transform infrared (FT-IR) spectroscopy using an ALPHA-P10098-4 spectrometer (Bruker Optics Inc., Billerica, MA, USA) in the ATR mode. Atomic force microscopy (AFM) was utilized to evaluate the surface morphology of the nanocomposites before and after elevated temperature exposure, with a Bruker Dimension Icon (Billerica, MA, USA). Samples were sputter-coated with 5 nm of platinum/palladium alloy to prevent charging of the nanocomposite before scanning electron microscopy (SEM) imaging (FESEM, model, JSM-7500, JEOL, JEOL; Tokyo, Japan). TEM samples were prepared by embedding coated polyethylene terephthalate into Epofix resin (EMS, Hatfield, PA, USA) and cured overnight in a silicone mold. The epoxy block was cut into 90 nm thick cross sections utilizing an Ultra 45° diamond knife (Diatome, Hatfield, PA). TEM micrographs were taken using a Tecnai G2F20 transmission electron microscope (FEI, Hillsboro, OR, USA), with an acceleration voltage of 200 kV. The nanocomposites degradation temperature (*T*_5%d_), which is where 5% of the sample’s weight is lost (excluding mass loss associated with water or solvent evaporation), was determined utilizing a Q-50 thermogravimetric analyzer (TA Instruments, New Castle, DE, USA). Approximately 3.4 mg of the nanocomposite was isothermally heated at 100 °C for 30 min to remove any residual moisture. The temperature was then increased at a constant rate of 10 °C min^–1^ up to 700 °C under a 60 mL min^–1^ flow of nitrogen. The nanocomposites reduced modulus (*E*_r_) and hardness (H) before and after elevated temperature exposure was assessed utilizing a TI 950 Triboindenter (Hysitron, Inc., Minneapolis, MN, USA) with a loading force of 200 μN to ensure indentation depth of ≈10%. A loading profile of 10 s of loading, 5 s at a stationary position, and 2 s of unloading was utilized. Surface wettability of the nanocomposite before and after elevated temperature exposure was evaluated utilizing a CAM 200 goniometer optical contact angle and surface tension meter (KSV Instruments, Ltd. Monroe, CT, USA). The nanocomposites were characterized by X-ray diffraction (XRD) using a diffractometer (BRUKER AXS model: D8 Discover) with copper K-alpha radiation equipped with a Vantec 500 2D detector. Samples were analyzed at a maximum power of 40 kV and 40 mA.

### Dielectric Properties Characterization

For dielectric characterization, nanocomposites were deposited on plasma-treated ITO-coated glass slides with a thickness of approximately 700 nm. All characterization occurred in ambient conditions unless specified. The breakdown strength (*E*_BD_) was determined utilizing a PolyK test fixture (Philipsburg, PA, USA) and a SCI 290 Hipot tester (Lake Forest, IL) as a DC voltage source. A breakdown event is defined as when a ≥1 mA current was detected. The contact electrode was a spring-loaded stainless-steel cap nut that made contact at constant pressure. Approximately 0.52 mm^2^ of the nanocomposite contacted the spring-loaded stainless-steel cap nut to ensure inhomogeneities did not influence the dielectric breakdown events. For elevated temperature testing, the test fixture was placed onto a hotplate and the temperature of the nanocomposite was monitored utilizing both a Fluke 64 MAX IR thermometer (Everett, Washington, USA) and a Fluke Thermocouple Thermometer utilizing a type K thermocouple. Prior to breakdown testing, the thermocouple was removed to prevent any short circuiting. Fifteen breakdown values were utilized for a Weibull probability of failure analysis to tabulate the nanocomposites’ *E*_BD_. Breakdown strength is defined as the breakdown strength at 63.2% of the probability of failure. Nanocomposite thickness and breakdown voltage values were utilized in conjunction to record *E*_BD_. A distance ≥3 mm separated each testing location to prevent previous breakdown events from influencing the next testing site. Stainless-steel cap nuts were changed out in between every five breakdown events to prevent tip corrosion from altering the nanocomposites’ breakdown strength. The dielectric constant (*k*) and loss was measured using a Keysight E4980AL/102 Precision LCR Meter (Keysight Technology, Santa Rosa, CA, USA). A gallium–indium eutectic compound was utilized as the top electrode contacting an area of approximately 1.0 mm^2^. Elevated temperature testing occurred by placing the sample onto a hot plate at the desired temperature.

### Thermal Conductivity Characterization

The through-plane thermal conductivity (*k*_⊥_) of the nanocomposites was measured by the 3ω technique using a custom-built setup, shown in Figure S2a.^22,23^ A shadow mask electron beam deposition technique was used to deposit an aluminum (Al) metal line in a four-probe pattern that acts both as a heater and sensor. The dimensions of the heater line were 4.7–5.0 mm long, 25–100 μm wide, and 250 nm thick. A 25 nm titanium (Ti) layer was deposited prior to deposition of an Al line to improve its adhesion. A schematic of the sample geometry is shown in Figure S2b. Validation of the 3ω system was performed by measuring the thermal conductivity of standard materials (fused quartz, Pyrex 7740, Si (undoped), and single-crystal sapphire (*c*-plane orientation) substrates) and is detailed in depth in a previous paper which uses the same measurement setup and procedure.^20^ In brief, each heater line was first calibrated by measuring the temperature dependence of resistance (d*R*/d*T*). The temperature coefficient of resistance of each Al sensor was in the range 0.002–0.003/K. The thermal conductivity of the standard substrates was 1.14 ± 0.05, 1.38 ± 0.06, 41.1 ± 1.6, and 146.5 ± 5.9 W m^–1^ K^–1^ for Pyrex 7740, fused quartz, sapphire, and silicon, respectively (at room temperature). The relative uncertainty in the measurement of these standards was within 4%, as shown in Table S1.

## Results and Discussion

### Breakdown Strength and Thermal Conductivity

The effective thermal conductivity of the nanocomposite, along with the thermal conductivity values of the bulk substrates (undoped silicon) and the nanocomposite at various thicknesses, were determined by fitting the experimental data [temperature amplitude (Δ*T*) vs current frequency (Hz)] to the data reduction method proposed by Tong et al. (see Figure S3a–c).^20,22^ The thermal conductivity of the underlying undoped silicon substrate was measured separately as a control and its value of 146.5 ± 5.9 W m^–1^ K^–1^ was used in data fitting. In order to determine the effective thermal conductivity of the nanocomposite, a series of films with varying thickness (*t*) were prepared and their thermal resistances (*R*) were obtained. The reported thermal resistances represent the film’s resistance as well as interfacial effects. The data in Table 1 present a direct relationship between thickness and thermal resistance (i.e., as thickness increases thermal resistance increases). Previous work, which compared experimental results to existing theoretical models, found that thermal boundary resistance at interfaces in LbL-assembled nanocomposites are the dominant factor governing film effective thermal conductivity.^20^ As thickness is increased, it is believed that the amount of interfaces (between platelets and the polymer matrix) also increases, leading to a lower thermal conductivity due to more interfacial thermal resistance. It is also possible that the intrinsic thermal conductivity of the film could vary as film thickness is increased due to subtle structural inhomogeneities hindering phonon transport.

**Table 1:** Thermal Resistance and Breakdown Strength at Various Thicknesses for the Dual Clay Nanocomposite

thickness (nm)	thermal resistance (*R*) (10^–^^7^ W m^–^^2^K^–^^1^)	breakdown strength (kV/mm)
280 ± 29	5	210
700 ± 10	23	249
1100 ± 100	58	240
*k*_eff_ 0.16 W m^–^^1^K^–^^1^		

From the obtained thermal resistance values at various thicknesses, the total thermal resistance (where resistance equals thickness divided by the thermal conductivity at said thickness) of the nanocomposite was calculated and the values were plotted against the film thickness (see Figure S3d). The effective thermal conductivity of the nanocomposite was then determined using the slope of the linear fit line in the resistance (*R*) as a function of thickness (*t*) plot. It is important to note that the term “effective thermal conductivity” refers to the thermal conductivity of the nanocomposite accounting for the presence of nonidealities and defects that can hinder thermal conductivity.^22^ It was determined that the dual clay nanocomposite had an effective thermal conductivity of 0.16 W m^–1^ K^–1^. From here on, when thermal conductivity is mentioned, it is referring to the effective thermal conductivity. It is important to note that the thermal conductivity of the nanocomposite at increasing thicknesses appears to converge on the effective thermal conductivity, suggesting that the data reduction method adequately accounts for nonideal effects in the nanocomposite.

Compared to the polymer matrix (PEI/PAA), which had a thermal conductivity of 0.46 W m^–1^ K^–1^, the dual clay nanocomposite demonstrated a 65% decrease in thermal conductivity. This reduced thermal conductivity is believed to be a consequence of the high interfacial density from the high inorganic loading, which creates a substantial amount of phonon scattering sites at the polymer-platelet interfaces.^24^ The thermal conductivity of the nanocomposite could also be hindered by the high loading of VMT (≈30 wt %), which is believed to have a low thermal conductivity and impressive electrical and thermal insulating properties.^25,26^ The nanocomposite’s breakdown strength remains relatively unchanged (210–250 kV/mm) in this thickness regime. This is likely due to a similar nanobrick wall microstructure at various thicknesses. It is believed that at even more elevated thicknesses, the nanocomposite’s breakdown strength may be slightly diminished due to the thickness effect (i.e., a higher occurrence of defect sites).^27^

**Figure 2:**
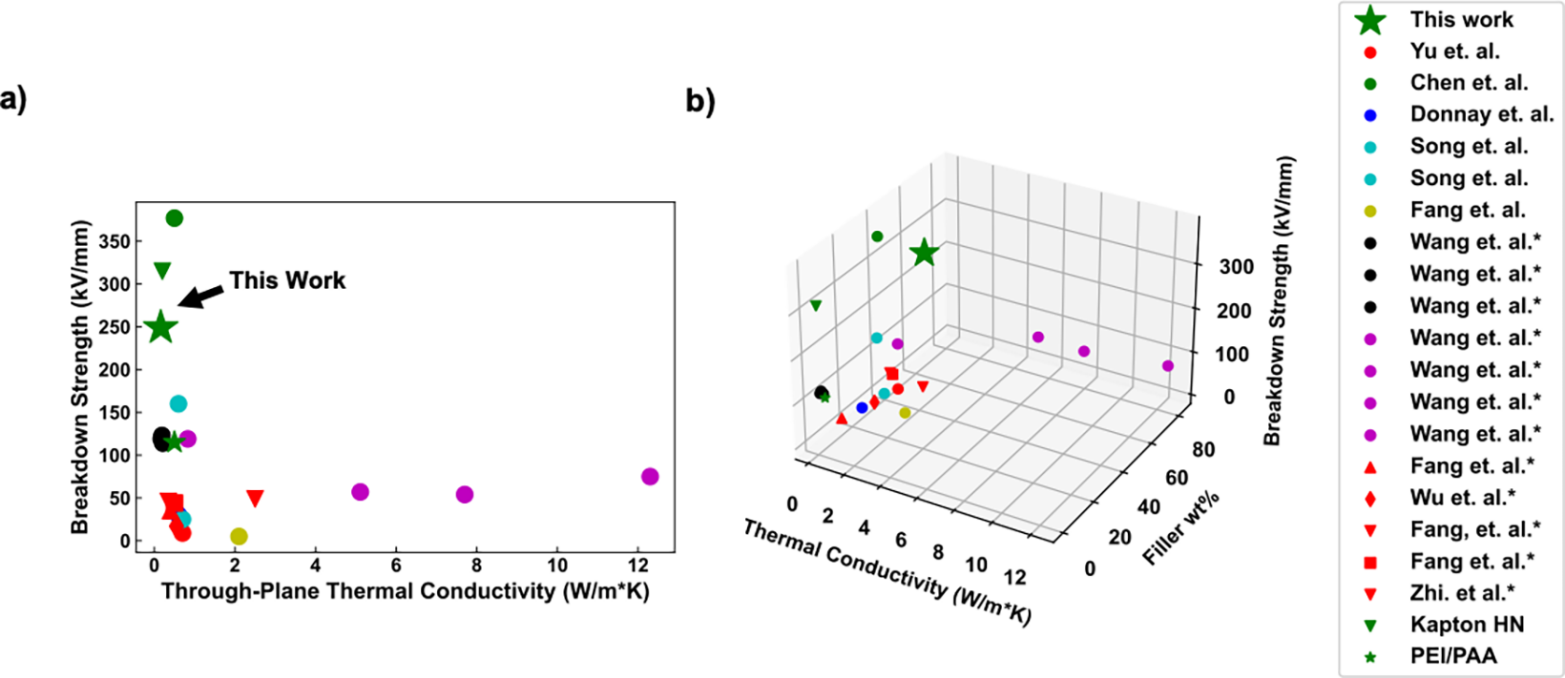
(a) Dielectric breakdown strength as a function of thermal conductivity and (b) dielectric breakdown strength as a function of thermal conductivity and filler wt % of various nanocomposites.^28−39^

The thermal conductivity, dielectric breakdown strength, and weight percent of filler in the nanocomposite reported herein, as well as nanocomposites in literature,^28−39^ are presented in Figure 2. For references with an asterisk next to them in the legend, it could not be determined if the thermal conductivity was through-plane or in-plane. The reported nanocomposite demonstrates comparable thermal conductivity and far superior breakdown strength than almost all of the systems displayed in Figure 2. There are limited reports of thermally conductive yet electrically insulating materials, as these properties are typically unobtainable with a single polymer-based material. These materials are difficult to achieve as thermal conductivity typically relies heavily on the transport of heat through both electron and phonon transportation. If a material is to be both thermally conductive and electrically insulating (i.e., have a high breakdown strength), the phonon contribution to thermal conductivity must be extraordinarily high and the electrical contribution negligible.^40^ The present dual clay nanocomposite demonstrates for the first time the dielectric and thermal transport properties of a polyelectrolyte-based nanocomposite.

### Dielectric Behavior

**Figure 3:**
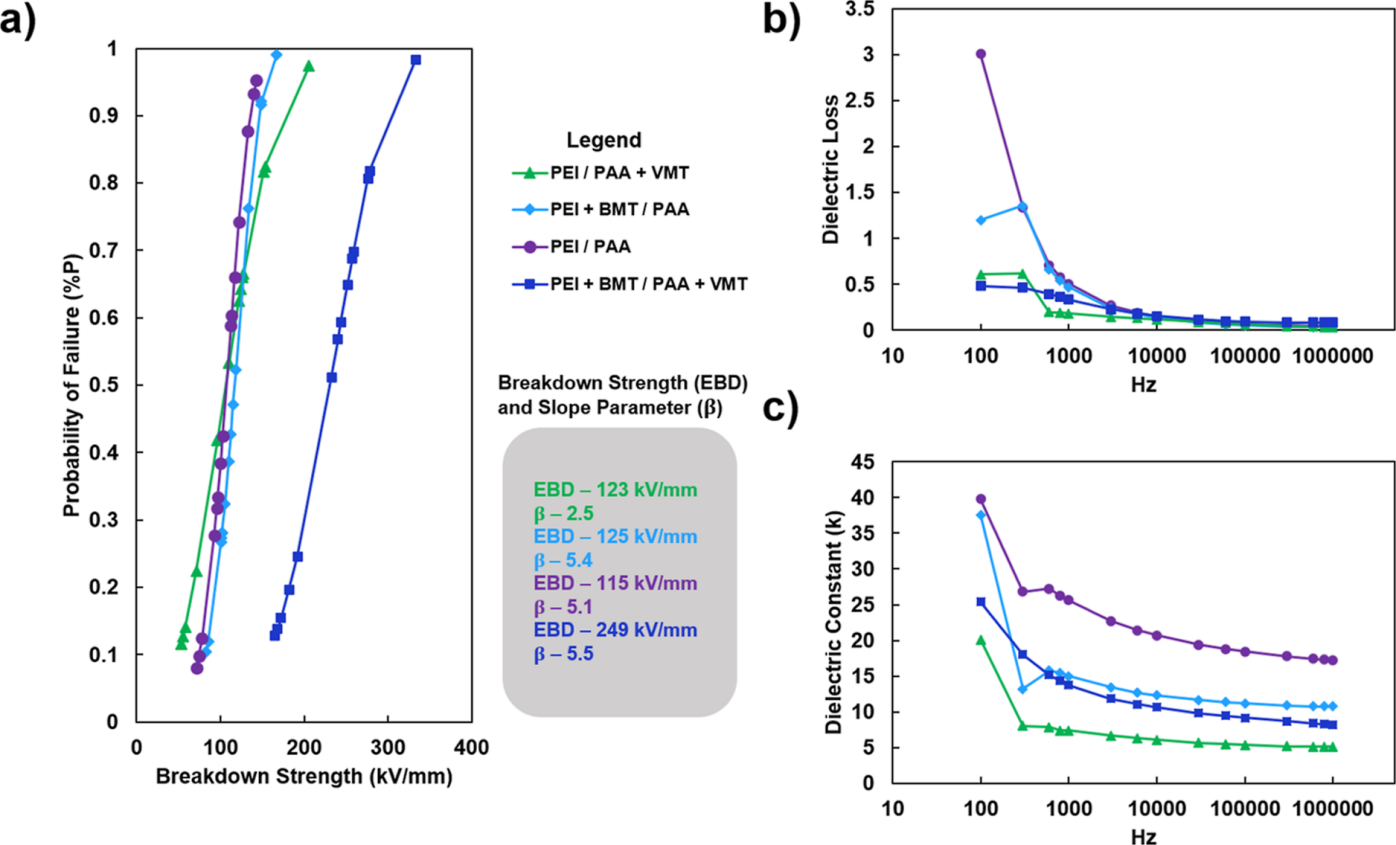
(a) Dielectric breakdown strength of systems with varying fillers, (b) dielectric loss of systems with varying fillers, from a frequency range of 100 Hz to 1 MHz, and (c) dielectric constant of systems with varying fillers, from a frequency range of 100 Hz to 1 MHz. A figure legend and color-coded dielectric breakdown strength (EBD) and slope parameters (β) are also provided.

The dielectric properties of the polymer matrix (PEI/PAA), single clay nanocomposites (PEI + BMT/PAA and PEI/PAA + VMT), and the dual clay nanocomposite (PEI + BMT/PAA + VMT) were investigated at a thickness of approximately 700 nm. This was done to minimize property change as a result of thickness variations.^27^ It was determined that PEI/PAA has the lowest breakdown strength (115 kV/mm), followed by the single clay systems, which have breakdown strengths of 123 kV/mm (PEI/PAA + VMT) and 125 kV/mm (PEI + BMT/VMT). It is believed that the inclusion of the platelets introduces a tortuous pathway for charge transport and therefore increases the breakdown strength of the nanocomposite. The dual clay nanocomposite has nearly double the breakdown strength (249 kV/mm) when compared to the single clay nanocomposite, which is believed to be a result of the significantly higher inorganic loading and the influence of a more tortuous pathway for charge transport. Having a high loading of inorganic material in polymer nanocomposites has been shown to greatly increase breakdown strength.^41,42^ It is important to note that some reports do show that breakdown strength can decrease as inorganic loading increases, but this typically occurs due to nanocomposite preparation techniques leading to more defects (e.g., filler aggregation or charge transport pathways), which can negatively impact breakdown strength.^43,44^ The dielectric properties of the analyzed systems are shown in Figure 3.

It is important to note that the terms loss %, dielectric loss, loss tangent, and tanδ are interchangeable with one another.^45−48^ When analyzing dielectric loss in the frequency regime of 100 Hz to 1 MHz, the PEI/PAA system demonstrates extraordinarily high losses compared to the other systems. These high losses and high dielectric constant (particularly in the realm of 100–1000 Hz) are to be expected of a polyelectrolyte multilayer system and can be attributed to ionic polarization as well as ion transport due to residual amounts of small ions in the film.^49^ After incorporating BMT or VMT independently into the nanocomposite, a significant decrease in the dielectric loss and constant occurs, especially in the realm of 100–1000 Hz. This decrease is believed to be a result of a tortuous pathway blocking small ion transport through the nanocomposite. A similar phenomenon is exploited in gas barrier and anti-corrosion nanocomposites.^50−52^ Priolo et al. reported that the oxygen transmission rate of nanobrick wall films significantly decreases as the inorganic loading increases due to the tortuous pathway.^52,53^ It is important to note that the dielectric constant of the PEI/PAA + VMT system is lower than any of the systems presented, most likely due to the nanocomposite being loaded with a clay that possesses a low degree of polarization, a phenomenon that has been known to lower a composite’s dielectric constant.^26,54^

Through incorporating both BMT and VMT into the nanocomposite, the dielectric loss is further reduced, likely due to a more tortuous pathway for ion transport. The nanostructure of this film can be seen in the cross-sectional TEM image in Figure S4. The dielectric constant of the dual clay containing nanocomposite (PEI + BMT/PAA + VMT) is between that of the PEI + BMT/PAA and PEI/PAA + VMT nanocomposites across the entire frequency range. It is also possible that the inclusion of BMT and VMT (independently and together), lowers the amounts of residual ions present in the nanocomposite which reduces the dielectric losses in the frequency range of 100–1000 Hz.

**Figure 4:**
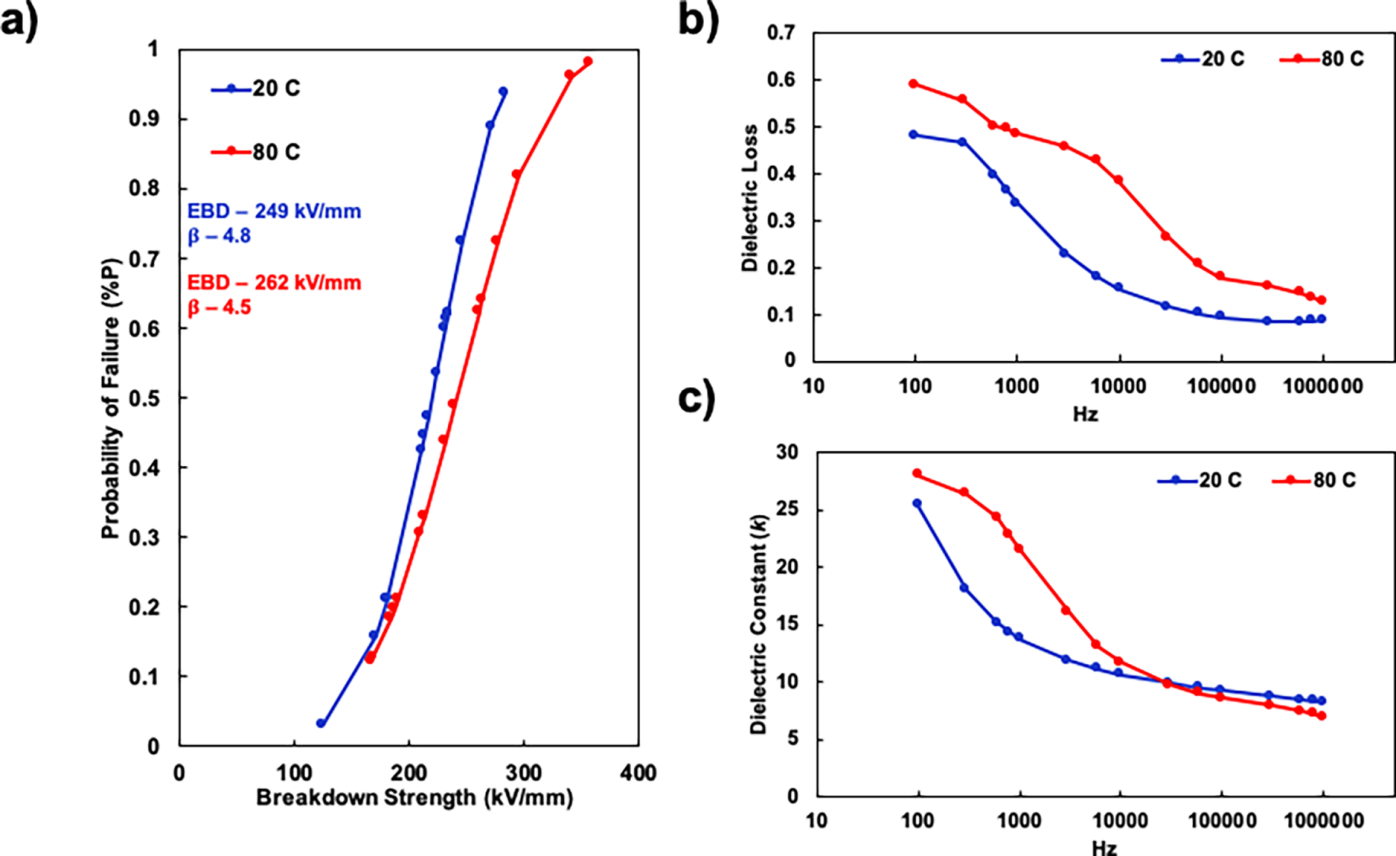
(a) Dielectric breakdown strength PEI + BMT/PAA + VMT at 20 and 80 °C, (b) dielectric loss at 20 and 80 °C, and (c) dielectric constant at 20 and 80 °C, from a frequency range of 100 Hz to 1 MHz.

The dielectric properties of the PEI + BMT/PAA + VMT nanocomposite were also investigated at elevated temperature (80 °C) to evaluate any changes in breakdown strength, dielectric constant, and dielectric loss. The surface morphology of the nanocomposite after room temperature and elevated temperature testing is presented in Figure S5. It was found that the dielectric constant and dielectric losses increase when exposed to elevated temperatures, which was expected due to most dielectric materials’ constant and losses increasing due to more energy available for charge transport, ionic polarization, and dipole movement.^55^ The dielectric breakdown strength of the nanocomposite increases from 249 to 262 kV/mm as testing temperature increases. Typically, as temperature increases, the dielectric breakdown strength of a material decreases as a result of thermally activated molecular, electronic, and ionic motion in the material.^56^ While this likely is occurring in the present system, it is believed that these effects are trumped by the expulsion of molecular water (discussed in the next section). Water is known to negatively impact (i.e., decrease) the breakdown strength of a material by promoting ionic and electronic transport.^57^ The dielectric properties of the PEI + BMT/PAA + VMT nanocomposite at 20 and 80 °C are presented in Figure 4.

### Mechanical Properties

**Figure 5:**
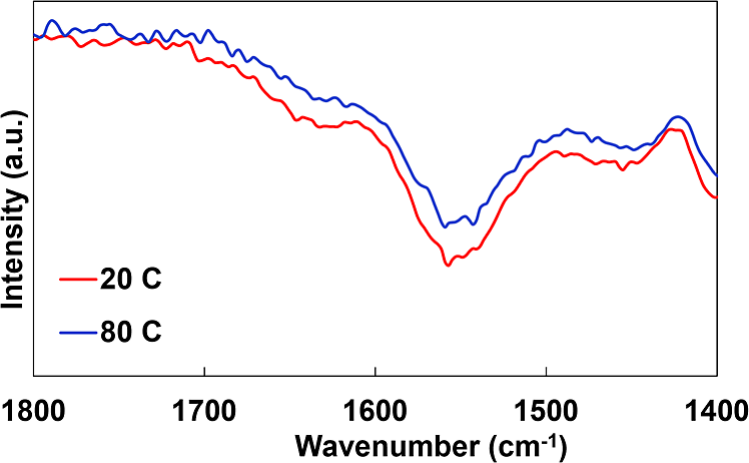
FT-IR spectra of the PEI + BMT/PAA + VMT nanocomposite after room temperature and 80 °C testing.

Immediately after elevated temperature dielectric testing, the characterization reported in this section occurred to ensure that minimal environmental moisture returned to the nanocomposite. It has been reported that thermally crosslinking PEI and PAA can increase the breakdown strength of a multilayer thin film.^13^ To confirm that the increase in breakdown strength was not a result of crosslinking, FT-IR was employed (Figure 5). It was found that there was no strong peak at 1640 cm^–1^, corresponding to an amide bond, after elevated temperature testing. Additionally, there was not a significant decrease or disappearance of a peak at approximately 1540 cm^–1^, a characteristic peak of PAA’s carboxylate. Through the lack of amide bond formation and carboxylate peak disappearance or reduction in intensity, thermal crosslinking was ruled out. It should be noted that significant thermal amidization takes hours to occur at low temperatures (<130 °C), so it is highly unlikely that elevated temperature testing resulted in amidization.^58^

Hariri et al. reported that decreasing water content in a polyelectrolyte complex will decrease thickness and increase modulus.^59^ This phenomenon is a result of water plasticizing/lubricating complexation sites and therefore decreasing modulus. The polyelectrolyte complex will conversely increase in thickness as water molecules will “swell” complexation sites. In order to measure the modulus (*E*_r_) and hardness (*H*) of the nanocomposites as a function of temperature exposure, without significant substrate influence, the nanocomposites were grown to a thickness of approximately five microns to ensure the “10% indention rule of thumb” could be employed.^60^ It was found that nanocomposites tested at elevated temperatures exhibit an increased modulus (from 6.8 GPa at 20 °C to 9.6 GPa at 80 °C) and an increased hardness (from 0.19 GPa at 20 °C to 0.24 GPa at 80 °C). This increased modulus and hardness are believed to be a result of molecular water expulsion. Table 2 summarizes the hardness and modulus changes.

**Table 2:** Hardness and Modulus of the Dual Clay Nanocomposite

exposure temperature (°C)	*H* (GPa)	*E*_r_ (GPa)
20	0.19 ± 0.12	6.8 ± 3.0
80	0.24 ± 0.14	9.6 ± 3.4

Along with increasing modulus and hardness values, the nanocomposites exhibit a ≈25% decrease in thickness after elevated temperature exposure. It is imperative to note that after elevated temperature testing, all nanocomposites experienced this reduction in film thickness, which is much lower than that reported for thermal crosslinking.^13,58^ The thickness reduction is dependent on humidity, but in all cases, film thickness decreased after elevated temperature testing. The nanocomposites’ water contact angle also increases from 32 to 39° after elevated temperature exposure, likely due to the removal of the hydration layer at the film surface. Figure S6 displays the water contact angle of the reported nanocomposites and PEI/PAA matrix. The presence of a hydration layer has been found to decrease polyelectrolyte-based films’ water contact angle.^61^ It is believed that an increase in dielectric breakdown strength is a result of molecular water expulsion at elevated temperatures. These findings are in good agreement with Kim and Shi’s work that demonstrated an increase in breakdown strength as modulus of a material increases.^62^

### Structure Characterization

**Figure 6:**
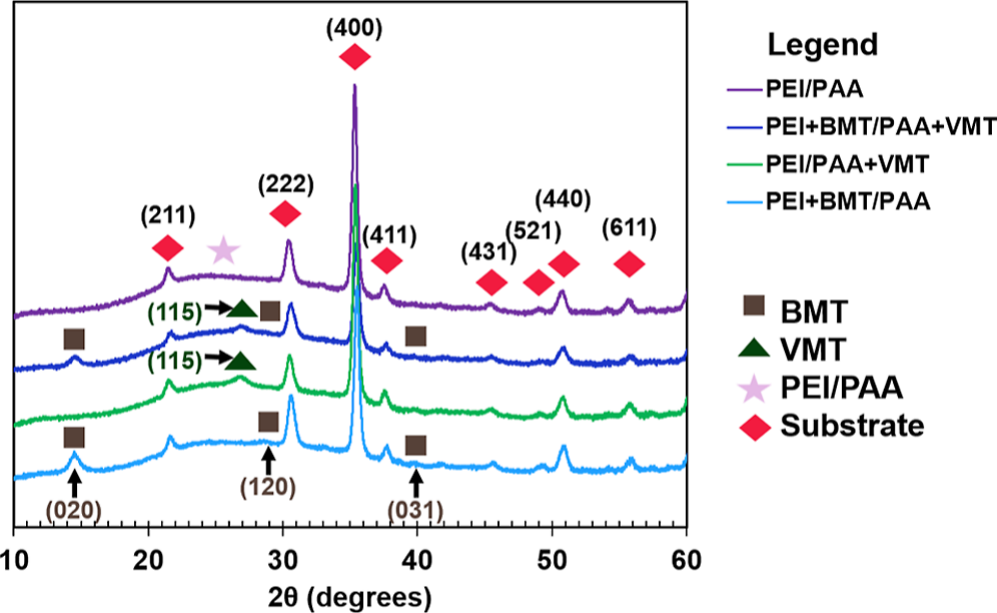
XRD spectra for systems with varying fillers.

XRD was performed on the presented systems to better understand their nanostructure (Figure 6). In the XRD spectra for all reported nanocomposites, peaks related to ITO can be observed as a result of the ITO-coated glass substrates. These peaks are represented by the red diamonds in Figure 6 and are in good agreement with literature values.^63,64^ In all spectra, a broad peak at approximately 25° signifies the amorphous structure of the PEI/PAA (i.e., polymer phase), which was expected as most PEI/PAA-based films are amorphous.^65^ It is believed that the low thermal conductivity (<0.5 W m^–1^ K^–1^) of the systems reported herein is greatly influenced by the nature of the PEI/PAA matrix, as semi-crystalline materials typically have lower thermal conductivity due to more phonon scattering. Figure S7 shows the XRD pattern of neat BMT powder, a drop-cast VMT film, and all analyzed systems. Neat BMT powder exhibits a basal reflection peak at 14.59° (020), which corresponds to a basal spacing of approximately 6.07 Å. This spacing agrees with the reported values in literature.^66^ The patterns for all BMT-containing nanocomposites show a similar peak, albeit slightly shifted to 14.52°, which indicates a basal spacing of approximately 6.10 Å. This minimal change in basal spacing suggests that the BMT platelets do not undergo a substantial amount of intercalation of polymer. Similarly, other characteristic peaks can be observed in the BMT-containing nanocomposites that match the BMT powder diffraction spectrum and reported values [i.e., (120) and (031)].^67^ Due to this minimal change, the structure of BMT is believed to not play a major role in altering the properties of the different nanocomposites. The XRD pattern for the drop-cast VMT film and the VMT-containing nanocomposites show a characteristic peak of VMT at approximately 26.67° (115), which matches that found in literature.^68,69^ In the drop-cast film, the VMT indicating peak shoulders a strong peak at 27.92°, which is believed to be a result of a possible surfactant that suspends VMT. It is important to note that this contamination peak is not reproduced in the VMT-containing systems. Similar to BMT, it is believed the structure of VMT plays a minimal role in altering the properties of the different nanocomposites, as its characteristic peak does not undergo any noticeable change. When relating structure to property, it is suspected that polymer and platelet crystallinity is not a dictating factor in the thermal conductivity decrease for the dual clay nanocomposite; it is believed that the number of polymer-platelet and platelet–platelet interfaces is causing an increase in phonon scattering, which is reducing the thermal conductivity of the nanocomposite.^20^ Furthermore, while the effects of BMT and VMT’s structure and crystallinity on the dielectric properties of the reported systems herein cannot be ruled out, it is believed that the improvement in dielectric properties is due to the significantly higher inorganic loading and the influence of a more tortuous pathway for charge transport when incorporating both platelets.

**Figure 7:**
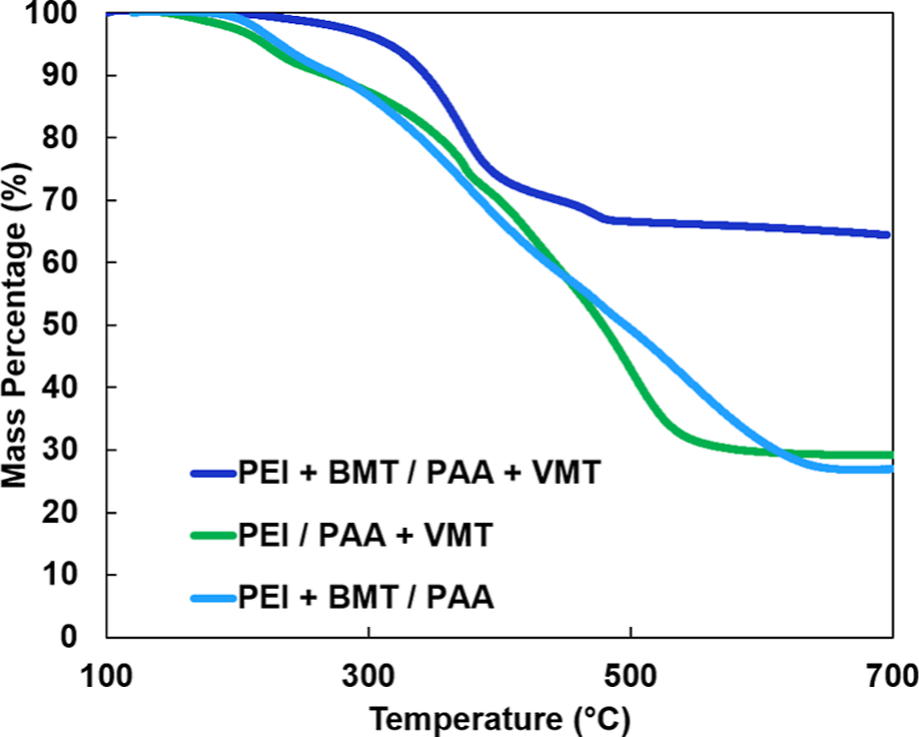
TGA thermographs of PEI + BMT/PAA, PEI/PAA + VMT, and PEI + BMT/PAA + VMT nanocomposites.

### Nanocomposite Thermal Resilience

The thermal resilience and inorganic loading weight percent of the nanocomposite were evaluated using thermogravimetric analysis (TGA), as shown in Figure 7. The PEI + BMT/PAA + VMT nanocomposite has an inorganic loading of 64 wt % and the PEI + BMT/PAA and PEI/PAA + VMT nanocomposites have inorganic loadings of 27 and 29 wt %, respectively. The PEI + BMT/PAA + VMT has more inorganic material due to both the cationic and anionic solutions having inorganic platelets. The degradation temperature (*T*_d5%_) is where 5% of the sample’s weight is lost (excluding mass loss associated with water or solvent evaporation). The PEI + BMT/PAA + VMT nanocomposite has a *T*_d5%_ of approximately 320 °C. This is significantly higher than the *T*_d5%_ of the PEI + BMT/PAA and PEI/PAA + VMT nanocomposites, which degrade at 230 and 220 °C, respectively. This higher *T*_d5%_ can be attributed to the significantly higher inorganic loading in the PEI + BMT/PAA + VMT nanocomposite. Higher inorganic loading in a nanocomposite has been known to increase the *T*_d5%_ of a nanocomposite regardless of its thermal conductivity.^70^

## Conclusions

This study is believed to be the first report of a polyelectrolyte-based nanocomposite utilized as a thermally conductive and electrically insulating nanodielectric. Through the inclusion of both BMT and VMT, the dielectric breakdown strength is increased by ≈115% when compared to the PEI/PAA matrix. This increase is believed to be due to the high inorganic loading creating a tortuous pathway in the nanocomposite for charge transport. The nanocomposite also boasts a high dielectric breakdown strength and reasonable through-plane thermal conductivity, a combination that is difficult to achieve simultaneously. Subjecting the nanocomposite to elevated temperatures was found to increase the breakdown strength, modulus, and hydrophobicity of the nanocomposite, which is attributed to the expulsion of molecular water in and on the surface of the nanocomposite. This report is the first LbL-generated nanocomposite that is shown to be electrically insulating and modestly thermally conductive. The findings outlined in this paper provide significant progress toward the creation of high-performance insulation systems for tomorrow’s high-voltage technologies.

## Supporting Information Available

Testing schematic for thermal conductivity, temperature vs amplitude and resistance vs thickness graphs, thermal conductivity of references, TEM micrographs, contact angle values, AFM and SEM images, and nanocomposite growth curve (PDF)
