# Supplementary material for: Nanobrick Wall Multilayer Thin Films with High Dielectric Breakdown Strength
Source: ACS Appl Eng Mater. 2023 Aug 18;1(9):2429–39. doi: 10.1021/acsaenm.3c00439 (PMC10862474; doi:10.1021/acsaenm.3c00439)
Supplement: Supplementary file 1 — em3c00439_si_008.pdf [file em3c00439_si_008.pdf]

Supporting Information

Nanobrick Wall Multilayer Thin Films with High Dielectric Breakdown Strength

Ethan T. Iverson,[a] Hudson Legendre,[b] Shubham V. Chavan,[c] Anil Aryal,[d] Maninderjeet Singh,[c] Sourav Chakravarty,[d] Kendra Schmieg,[b] Hsu-Cheng Chiang,[a] Carolyn T. Long,[b] Patrick J. Shamberger,[d] Alamgir Karim,[c] and Jaime C. Grunlan[abd]*

[a] Department of Chemistry, Texas A&M University, College Station, TX 77843, USA

[b] Department of Mechanical Engineering, Texas A&M University, College Station, TX 77843, USA

[c] Department of Chemical and Biomolecular Engineering, University of Houston, Houston, TX, 77204,USA

[d] Department of Materials Science and Engineering, Texas A&M University, College Station, TX 77843, USA

* Corresponding author e-mail: jgrunlan@tamu.edu

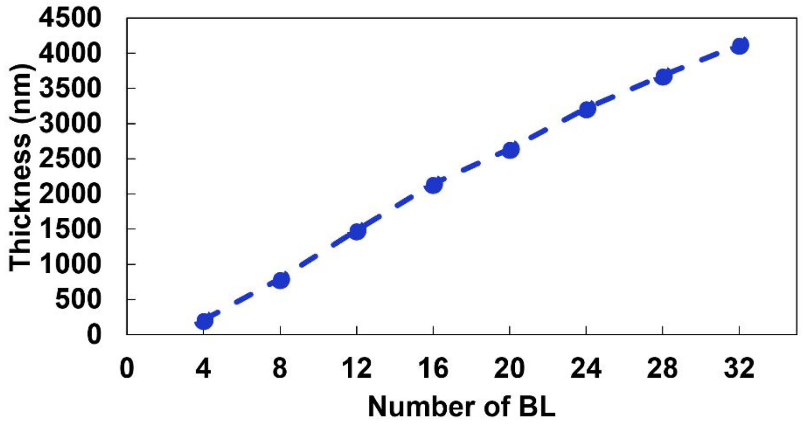

Figure S1. Growth curve for the PEI+BMT/PAA+VMT nanocomposite.

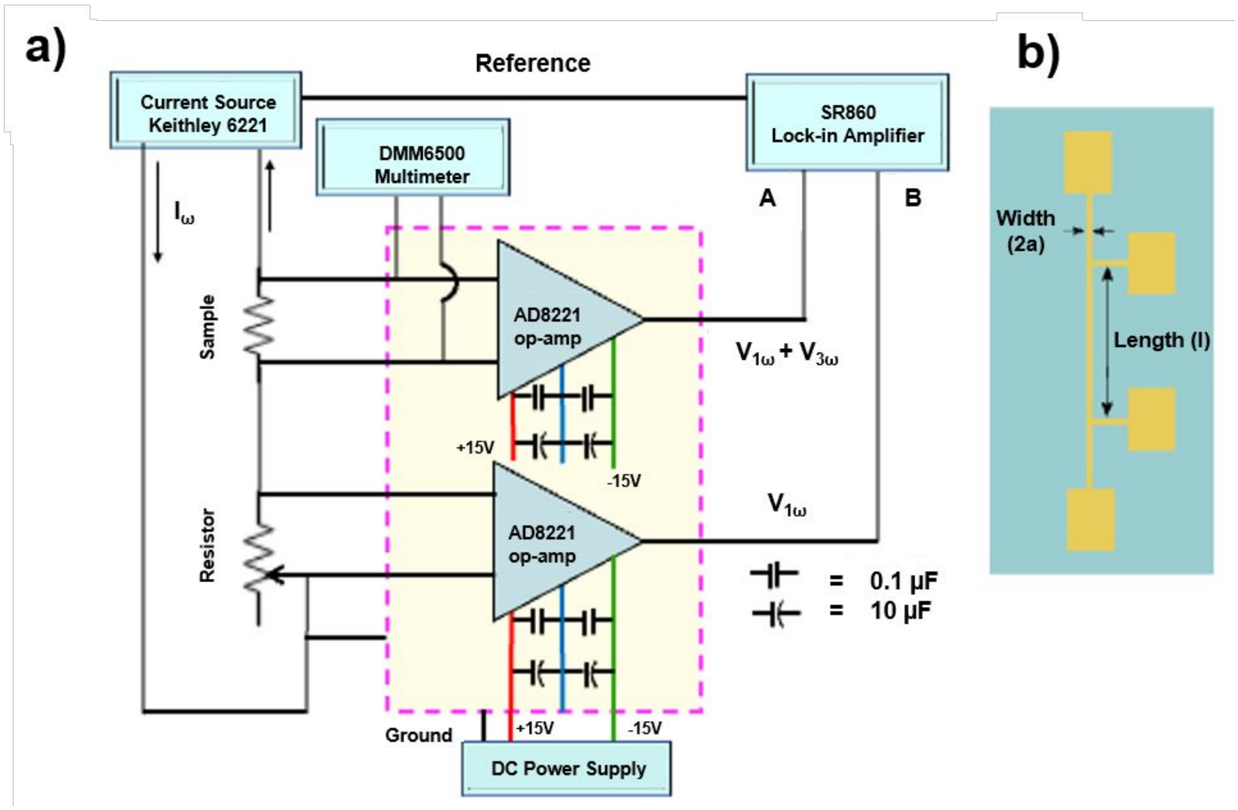

Figure S2. (a) Schematics of the custom built 3-omega setup. The AC current (I_{ω}) is generated by a Keithley 6221 current source. A variable resistor is connected in series to compensate the first harmonic voltage ($V_{1\omega}$) from the sample. The third harmonic voltage ($V_{3\omega}$) is measured by the lock-in amplifier (SR 860). (b) The 3ω sample geometry.

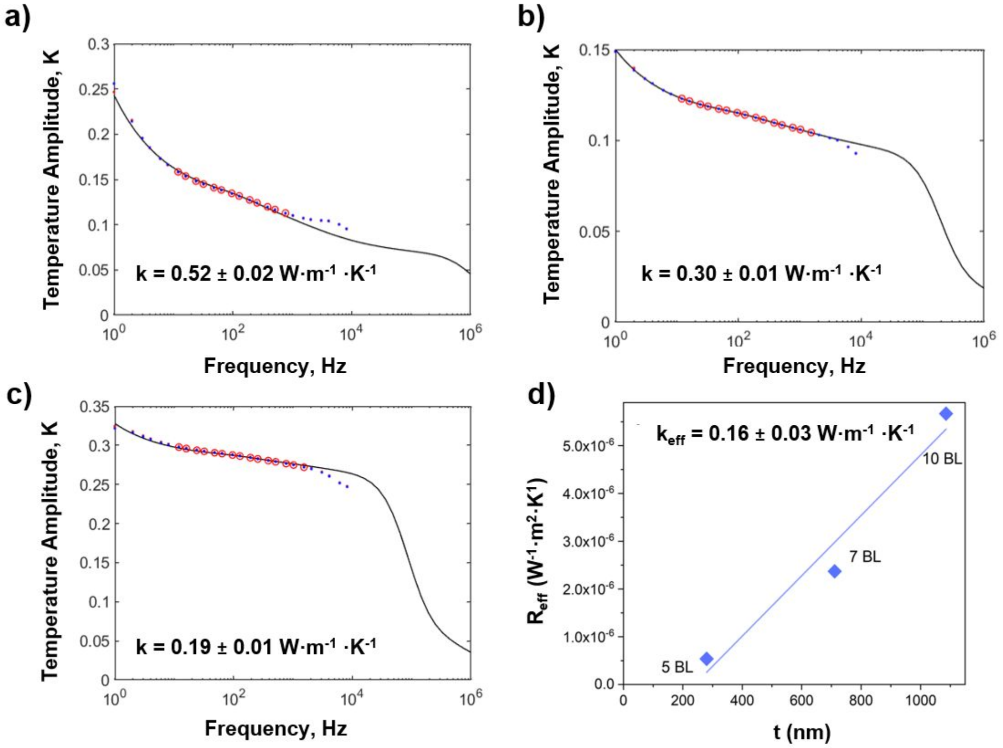

Figure S3. Temperature amplitude vs frequency for (a) 5 BL (b) 7 BL, and (c) 10 BL 0.1 wt% PEI 0.5 wt% BMT/ 0.1 wt% PAA 1 wt% VMT nanocomposite films. The solid line is the theoretical model, solid dots are the measured values, and red open circles are fitted data points to the model. (d) Thickness dependence of total thermal resistance of film.

Table S1. Measured and reported values of thermal conductivity of standard substrates.

Material	Measured k ($W \cdot m^{-1} \cdot K^{-1}$)	Reported k ($W \cdot m^{-1} \cdot K^{-1}$)	References
Fused Quartz	1.38 ± 0.06	1.3 – 1.39	1, 2-4
Pyrex 7740	1.14 ± 0.05	1.11 – 1.26	1, 5-8
Sapphire	41.1 ± 1.6	34.84 – 42.65	9, 10
Silicon	146.5 ± 5.9	128 – 156	11-15

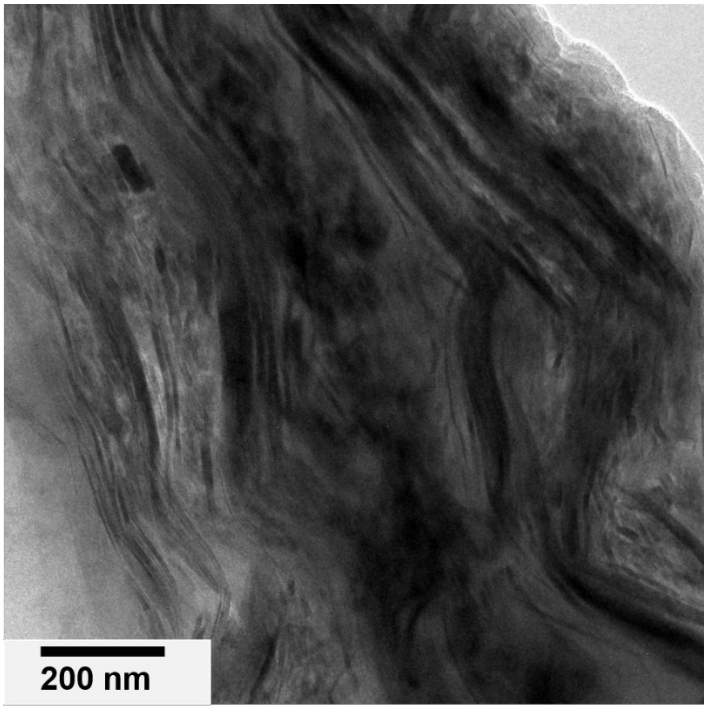

Figure S4. Cross sectional transmission electron micrograph of the PEI+BMT/PAA+VMT nanocomposite.

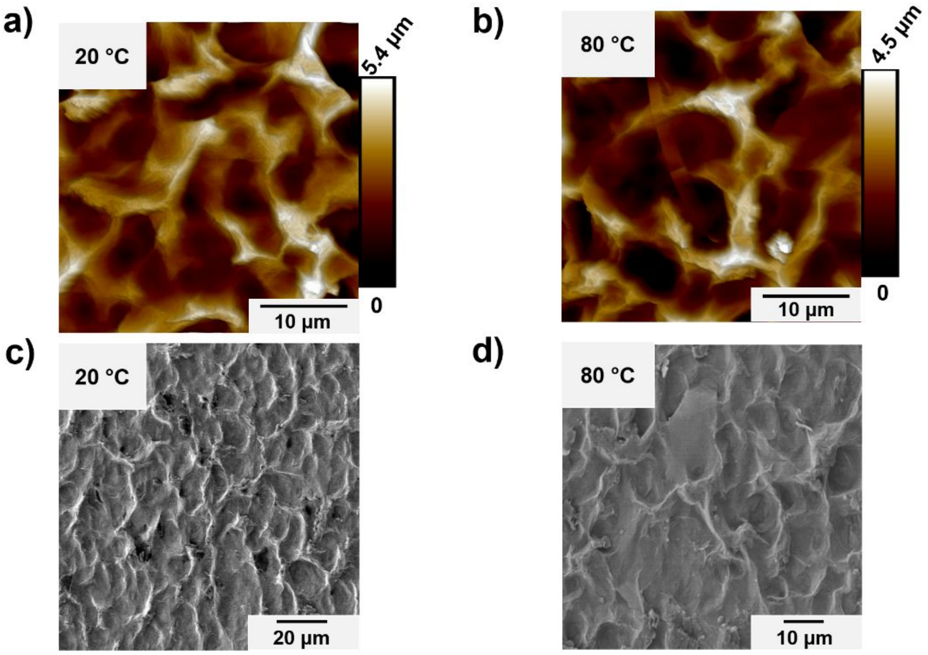

Figure S5. Morphology of the nanocomposite before (a,c) and after (b,d) elevated temperature exposure, evaluated with AFM (a,b) and SEM (c,d).

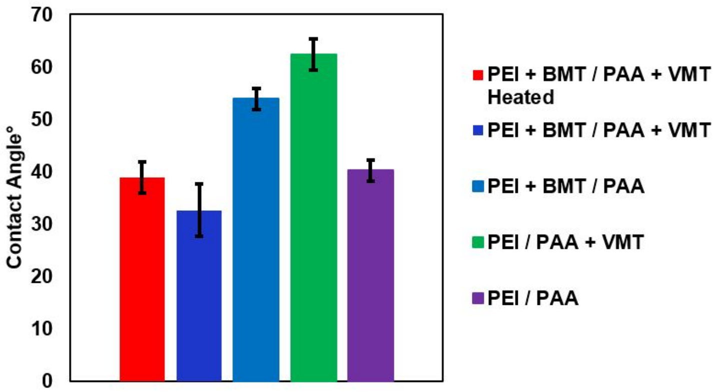

Figure S6. Water contact angles of nanocomposites and the PEI/PAA matrix.

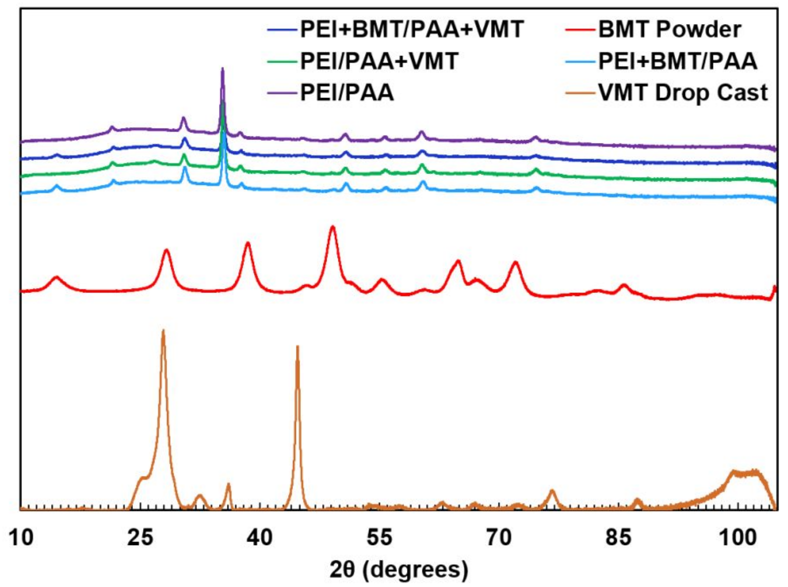

Figure S7. X-ray diffraction spectra of BMT powder, a VMT drop cast film, and systems with varying fillers.

REFERENCES:

(1) Cahill, D. G. Thermal Conductivity Measurement from 30 to 750 K: The 3ω Method. *Review of Scientific Instruments* **1990**, *61* (2), 802–808. https://doi.org/10.1063/1.1141498.

(2) Oh, D.-W.; Jain, A.; Eaton, J. K.; Goodson, K. E.; Lee, J. S. Thermal Conductivity Measurement and Sedimentation Detection of Aluminum Oxide Nanofluids by Using the 3ω Method. *International Journal of Heat and Fluid Flow* **2008**, *29* (5), 1456–1461. https://doi.org/10.1016/j.ijheatfluidflow.2008.04.007.

(3) Sugawara, A. Precise Determination of Thermal Conductivity of High Purity Fused Quartz from 0° to 650°C. *Physica* **1969**, *41* (3), 515–520. https://doi.org/10.1016/0031-8914(69)90053-6.

(4) Kimling, J.; Martens, S.; Nielsch, K. Thermal Conductivity Measurements Using 1ω and 3ω Methods Revisited for Voltage-Driven Setups. *Review of Scientific Instruments* **2011**, *82* (7), 074903. https://doi.org/10.1063/1.3606441.

(5) Yang, G.; Migone, A. D.; Johnson, K. W. Heat Capacity and Thermal Diffusivity of a Glass Sample. *Phys. Rev. B* **1992**, *45* (1), 157–160. https://doi.org/10.1103/PhysRevB.45.157.

(6) Assael, M. J.; Gialou, K.; Kakosimos, K.; Metaxa, I. Thermal Conductivity of Reference Solid Materials. *International Journal of Thermophysics* **2004**, *25* (2), 397–408. https://doi.org/10.1023/B:IJOT.0000028477.74595.d5.

(7) Assael, M. J.; Antoniadis, K. D.; Metaxa, I. N.; Mylona, S. K.; Assael, J.-A. M.; Wu, J.; Hu, M. A Novel Portable Absolute Transient Hot-Wire Instrument for the Measurement of the Thermal Conductivity of Solids. *Int J Thermophys* **2015**, *36* (10), 3083–3105. https://doi.org/10.1007/s10765-015-1964-6.

(8) Gaal, P.; Thermitus, M.-A.; Stroe, D. Thermal Conductivity Measurements Using the Flash Method. *Journal of Thermal Analysis and Calorimetry* **2005**, *78* (1), 185–189. https://doi.org/10.1023/b:jtan.0000042166.64587.33.

(9) Cahill, D. G.; Lee, S.-M.; Selinder, T. I. Thermal Conductivity of κ-Al2O3 and α-Al2O3 Wear-Resistant Coatings. *Journal of Applied Physics* **1998**, *83* (11), 5783–5786. https://doi.org/10.1063/1.367500.

(10) Chernodoubov, D. A.; Inyushkin, A. V. Automatic Thermal Conductivity Measurements with 3-Omega Technique. *Review of Scientific Instruments* **2019**, *90* (2), 024904. https://doi.org/10.1063/1.5084103.

(11) Lee, Y.; Hwang, G. S. Mechanism of Thermal Conductivity Suppression in Doped Silicon Studied with Nonequilibrium Molecular Dynamics. *Phys. Rev. B* **2012**, *86* (7), 075202. https://doi.org/10.1103/PhysRevB.86.075202.

(12) Kim, J. H.; Feldman, A.; Novotny, D. Application of the Three Omega Thermal Conductivity Measurement Method to a Film on a Substrate of Finite Thickness. *Journal of Applied Physics* **1999**, *86*, 3959–3963. https://doi.org/10.1063/1.371314.

(13) Wilson, R. B.; Cahill, D. G. Limits to Fourier Theory in High Thermal Conductivity Single Crystals. *Appl. Phys. Lett.* **2015**, *107* (20), 203112. https://doi.org/10.1063/1.4935987.

(14) Braun, J. L.; Olson, D. H.; Gaskins, J. T.; Hopkins, P. E. A Steady-State Thermoreflectance Method to Measure Thermal Conductivity. *Review of Scientific Instruments* **2019**, *90* (2), 024905. https://doi.org/10.1063/1.5056182.

(15) Glassbrenner, C. J.; Slack, G. A. Thermal Conductivity of Silicon and Germanium from 3°K to the Melting Point. *Phys. Rev.* **1964**, *134* (4A), A1058–A1069. https://doi.org/10.1103/PhysRev.134.A1058.
